# Supplementary material for: Identification and Characterization of a Novel Hypovirus from the Phytopathogenic Fungus Botryosphaeria dothidea
Source: Viruses. 2023 Oct 7;15(10):2059. doi: 10.3390/v15102059 (PMC10611357; doi:10.3390/v15102059)
Supplement: Supplementary file 1 [file viruses-15-02059-s001.zip › Supplementary Table S1.pdf]

Supplementary Table S1. Primers used in this study.

Primer Name	Oligonucleotide sequence (5′- 3′)	Usage
BdHV1deF2	TCACACGGTCGTGACAGAAG	For detecting BdHV1
BdHV1deR2	GACAGTCCAGCGTGGTTACA	
BdCV1-CPdeF	GGGAACCAGAGCACGATTCA	For detecting BdCV1
BdCV1-CPdeF	ACGGTGAAGTTGTCGATCC	
BdPV1-RdRpdeF	CCCTCCGGATCTGGTTTCAC	For detecting BdPV1
BdPV1-RdRpdeF	GGGATTTCGTCCAGTCCGAG	
BdVV3-RdRpdeF	CAGCAGCCAACAAGGTCAAC	For detecting BdVV3
BdVV3-RdRpdeF	GCCGGTATTCACGATCGACT	
BdPmV1-RdRpdeF	ACGATTTCCATCAGCCACTAC	For detecting BdPmV1
BdPmV1-RdRpdeR	CGGCGAGACGTTGAATATGA	
BdHV1-F1	GGTGCAACTTCACAGCCTTG	For cloning of BdHV1/SXD111
BdHV1-R1	TGACAGTCCAGCGTGGTTAC	
BdHV1-F2	CCTTTTCGGCTGTTGGTGTG	
BdHV1-R2	ACCAGTCAGCTTCTGTCACG	
BdHV1-F3	CGAACGACCCCTTGTGAAGA	
BdHV1-R3	CCAGCTATTTCACGCGCTTC	
BdHV1-F4	TTTTCTGTCGCCCCGCAT	
BdHV1-R4	TGCGCAACTCGAACCTGT	
BdHV1-F5	TGTTTCACTGTCTCGACCCG	
BdHV1-R5	TCTTCACAAGGGGTCGTTCG	
BdHV1-F6	ATGCGCCCTTTCAAACGC	
BdHV1-R6	AGCGTGCGCCAAGTAGAA	

BdHV1-F7	TTGCAGCGCTCCATGACA	
BdHV1-R7	ACGGCCGTTGTCAAACCA	
BdHV1-F8	AGCGGCATGGAATCTCGG	
BdHV1-R8	TGCTGGGGCGCTTGATAC	
BdHV1-3RACE1	CGTCGAGCTCGATGTTCAG	For terminal cloning of BdHV1/SXD111
BdHV1-3RACE2	GGGCAAAGCTGTTACTTG	
BdHV1-5RACE1	GCCACTTGTGAGAGGTACG	
BdHV1-5RACE2	GGAAGAATTGGGCTTAACG	
RACE-OLIGO	GCATTGCATCATGATCGATCGAATTCTTTAGTGAGGGTTAA TTGCC	For RLM-RACE of BdPmV1 and BdPV1
RACE-1st	GGCAATTAACCCTCACTAAAG	For terminal cloning of BdPmV1 and BdPV1
RACE-2nd	TCACTAAAGAATTCGATCGATC	
RACE-3rd	CGATCGATCATGATGCAATGC	
BdPmV1RNA1F	TCCGCCCACTACTCAACAAC	For cloning of BdPmV1-dsRNA1
BdPmV1RNA1R	GTCCACGTCAACCATCGAGT	
BdPmV1RNA1-3RACE	GGCTATTGACAAGCTCTAC	For terminal cloning of BdPmV1-dsRNA1
BdPmV1RNA1-5RACE	CGGCGAGACGTTGAATATG	
BdPmV1RNA2F	GGAGTCAGCCATGCTCTTGT	For cloning of BdPmV1-dsRNA2
BdPmV1RNA2R	TGTGAGTGATGACGTCTGCC	
BdPmV1RNA2-3RACE	CGTCAGTGTGGATGATTACC	For terminal cloning of BdPmV1-dsRNA2
BdPmV1RNA2-5RACE	GTGGACACTACCTCAAGTATTCG	
BdPmV1RNA3F	CCGTTCAACA TCTTCGAGTTC	For cloning of BdPmV1-dsRNA3
BdPmV1RNA3R	GGAGTTGATCATGAACCCAG	
BdPmV1RNA3-3RACE	CCATGCACTTCGTCGCCAG	For terminal cloning of BdPmV1-dsRNA3
BdPmV1RNA3-5RACE	GCGTGGGAGTATTTGGAG	

BdPmV1RNA4-3RACE	CCATAGGTCCCGGGGTTGGAG	For terminal cloning of BdPmV1-dsRNA4
BdPmV1RNA4-5RACE	TCATCAAGTTGGCGTCCCTC	
BdPmV1RNA5-3RACE	GTCTTGGCGAGCTGGTAGTAG	For terminal cloning of BdPmV1-dsRNA5
BdPmV1RNA5-5RACE	GGAGGACATTGAGTTCGAGATC	
BdPV1RNA1-3RACE	GGGATTTCGTCCAGTCCGAG	For terminal cloning of BdPV1-dsRNA1
BdPV1RNA1-5RACE	CCCTCCGGATCTGGTTTCAC	
BdPV1RNA2-3RACE	CCGTCAAACCAAGCCTCC	For terminal cloning of BdPV1-dsRNA2
BdPV1RNA2-5RACE	CGAAGCGATCGACGAGATG	
BdPV1RNA3-3RACE	GGCTAGTCGAACGTAGGAGC	For terminal cloning of BdPV1-dsRNA3
BdPV1RNA3-5RACE	CTTCCGCAGTCGGGTAAGAG	
RdPdN6	CGATCGATCATGATGCAATGCNNNNNN	For random cloning of BdPV1/SXD111-dsRNA3
RdSP	CGATCGATCATGATGCAATGC	
