# Supplementary material for: Identification and Characterization of a Novel Hypovirus from the Phytopathogenic Fungus Botryosphaeria dothidea
Source: Viruses. 2023 Oct 7;15(10):2059. doi: 10.3390/v15102059 (PMC10611357; doi:10.3390/v15102059)
Supplement: Supplementary file 1 [file viruses-15-02059-s001.zip › Supplementary Table S2.pdf]

Supplementary Table S2. Abbreviations of virus names and viral polyprotein accession numbers used in alignment analysis in this study.

Virus	Abbreviation	accession number
Alphahypovirus		
Cryphonectria hypovirus 1	CHV1	AAA67458
Cryphonectria hypovirus 2	CHV2	AAA20137
Betahypovirus		
Cryphonectria hypovirus 3	CHV3	AAF13604
Cryphonectria hypovirus 4	CHV4	AAQ76546
Sclerotinia sclerotiorum hypovirus 1	SsHV1	AE699352
Setosphaeria turcica hypovirus 1	StHV1	AZT88613
Trichoderma harzianum hypovirus 1	ThHV1	QGA30969
Fusarium oxysporum dianthi hypovirus 2	FodHV2	QHI00074
Valsa ceratosperma hypovirus 1	VcHV1	BAM08994
Phomopsis longicolla hypovirus	PlHV1	AIG94930
Botrytis cinerea hypovirus 1	BcHV1	WAT23236
Gammahypovirus		
Sclerotium rolfsii hypovirus 2	SrHV2	AZF86107
Sclerotium rolfsii hypovirus 3	SrHV3	AZF86108
Deltahypovirus		
Beihai sipunculid worm virus 6	BHSWV6	APG76084
Beihai hypo-like virus 1	BH-LV1	APG76085
Epsilonhypovirus		
Agaricus bisporus virus 2	AbV2	AQM49947
Fusarium poae hypovirus 1	FpHV1	BAV56305
Zetahypovirus		
Sclerotinia sclerotiorum hypovirus 6	SrHV6	AZF86111
Etahypovirus		
Sclerotium rolfsii hypovirus 8	SrHV8	AZF86113
Thetahypovirus		
Sclerotium rolfsii hypovirus 1	SrHV1	AZA15168
Sclerotinia sclerotiorum hypovirus 2	SsHV2	QBA69886
