# Supplementary material for: Identification and Characterization of a Novel Hypovirus from the Phytopathogenic Fungus Botryosphaeria dothidea
Source: Viruses. 2023 Oct 7;15(10):2059. doi: 10.3390/v15102059 (PMC10611357; doi:10.3390/v15102059)
Supplement: Supplementary file 1 [file viruses-15-02059-s001.zip › Supplementary Table S3.pdf]

Supplementary Table S3. Amino acid identity between BdHV1/SXD111 and representative members of the genus *Hypovirus* in the polyprotein.

Virus name	Length (aa)	Coverage (%)	E value	Identity (%)	Accession number
Cryphonectria hypovirus 4	2848	77	0	49.77	YP_138519
Alternaria dianthicola hypovirus 1	2745	74	0	49.70	UYZ32447
Erysiphe necator associated hypovirus 1	3522	75	0	48.05	QHD64829
Phomopsis longicolla hypovirus	2848	77	0	48.12	YP_009051683
Trichoderma harzianum hypovirus 1	2807	74	0	49.39	QGA30970
Cryphonectria hypovirus 3	2874	77	0	47.65	AAF13603
Valsa ceratosperma hypovirus 1	2940	74	0	48.11	YP_005476604
Monilinia fructicola hypovirus 1	2918	78	0	46.46	UTQ48841
Sclerotinia sclerotiorum hypovirus 7	2973	74	0	48.22	QUE49155
Botrytis cinerea hypovirus 1	2965	74	0	48.14	UNI72644
Fusarium concentricum hypovirus 1	2950	77	0	46.73	BCP96877
Botrytis cinerea hypovirus 3	3042	77	0	45.93	QJT73707
Botrytis cinerea hypovirus 5a	2972	78	0	45.88	WGO62045
Sclerotinia sclerotiorum hypovirus 1	2948	78	0	45.29	YP_004782527
Fusarium oxysporum dianthi hypovirus 2	2892	74	0	46.61	YP_010800030
Setosphaeria turcica hypovirus 1	2751	71	0	49.79	YP_010799558
